# Risk Stratification for Diabetic Retinopathy Screening Order Using Deep Learning: A Multicenter Prospective Study

**DOI:** 10.1167/tvst.12.12.11

**Published:** 2023-12-11

**Authors:** Ashish Bora, Richa Tiwari, Pinal Bavishi, Sunny Virmani, Rayman Huang, Ilana Traynis, Greg S. Corrado, Lily Peng, Dale R. Webster, Avinash V. Varadarajan, Warisara Pattanapongpaiboon, Reena Chopra, Paisan Ruamviboonsuk

**Affiliations:** ^1^ Google Health, Palo Alto, CA, USA; ^2^ Department of Ophthalmology, College of Medicine, Rangsit University, Rajavithi Hospital, Bangkok, Thailand; ^3^ Work done at Google via Advanced Clinical, Deerfield, IL, USA

**Keywords:** deep learning, diabetic retinopathy progression, real world implementation, DR progression model, risk stratification

## Abstract

**Purpose:**

Real-world evaluation of a deep learning model that prioritizes patients based on risk of progression to moderate or worse (MOD+) diabetic retinopathy (DR).

**Methods:**

This nonrandomized, single-arm, prospective, interventional study included patients attending DR screening at four centers across Thailand from September 2019 to January 2020, with mild or no DR. Fundus photographs were input into the model, and patients were scheduled for their subsequent screening from September 2020 to January 2021 in order of predicted risk. Evaluation focused on model sensitivity, defined as correctly ranking patients that developed MOD+ within the first 50% of subsequent screens.

**Results:**

We analyzed 1,757 patients, of which 52 (3.0%) developed MOD+. Using the model-proposed order, the model's sensitivity was 90.4%. Both the model-proposed order and mild/no DR plus HbA1c had significantly higher sensitivity than the random order (*P* < 0.001). Excluding one major (rural) site that had practical implementation challenges, the remaining sites included 567 patients and 15 (2.6%) developed MOD+. Here, the model-proposed order achieved 86.7% versus 73.3% for the ranking that used DR grade and hemoglobin A1c.

**Conclusions:**

The model can help prioritize follow-up visits for the largest subgroups of DR patients (those with no or mild DR). Further research is needed to evaluate the impact on clinical management and outcomes.

**Translational Relevance:**

Deep learning demonstrated potential for risk stratification in DR screening. However, real-world practicalities must be resolved to fully realize the benefit.

## Introduction

Diabetic retinopathy (DR), a complication of diabetes, is a major cause of visual loss globally.^1^ The diabetic patient population is expected to grow from 415 million in 2015 to 642 million in 2040, with an expected concomitant increase in DR incidence.^2^ Regular screening is key to preventing irreversible blindness caused by DR,^3^ and several countries worldwide have implemented screening programs that use color fundus photography (CFP) to identify those at risk of developing sight-threatening DR.^4^

However, DR screening faces a scaling challenge as the number of patients with diabetes rises.

Of those with diabetes, 75% are in low-to-middle income countries, and the burden of diabetes and its related conditions disproportionately affects these populations.^5^ Relatedly, low-to-middle income countries are more likely to suffer from constraints in resources and trained health care professionals.^6^ The coronavirus disease 2019 (COVID-19) pandemic added further strain to health care systems, and nonurgent medical appointments for DR screenings were deferred for several months. With the resumption of screenings, hospitals and clinics experienced backlogs and were overburdened with the consequences of delayed care and screening.^7^^–^^10^

These challenges may be addressed by optimizing screening intervals personalized to a patient's risk of DR progression, potentially improving program efficiency and vision-related outcomes while also reducing cost. In recent times, deep learning (DL) systems have been applied to automated computational grading of fundus photos for DR assessment, and many have shown expert-level accuracy.^11^^–^^14^ However, few studies have investigated the application of DL for DR risk stratification to help optimize DR screening intervals or identify those at most urgent need of screening. A previous study developed and retrospectively validated a DL model that uses CFPs to predict the risk of developing mild or worse DR in patients with diabetes without DR.^15^ This DL system provides a score that indicates the predicted likelihood of developing DR within the next two years. The model's area under the curve for predicting development of DR across two validation sets were 0.79 and 0.70 (95% confidence intervals, 0.77–0.81 and 0.67–0.74, respectively).

In the present study, we conducted a prospective evaluation of the clinical usefulness of the aforementioned DR progression model to prioritize patients according to their risk of progression based on prior CFPs. This study was conducted in Thailand during the COVID-19 pandemic, which significantly impacted the country's national DR screening program—a program that serves an estimated 4.5 million patients with diabetes.^16^ The DR progression model was extended to predict the likelihood of progression from no or mild DR to referable disease defined as moderate or worse (MOD+) DR (Early Treatment Diabetic Retinopathy Study level ≥43) within 1 year. We hypothesized that the model would aid in prioritizing patients for prompt screening and deferring appointments for nonurgent cases in case of capacity constraints. In this nonrandomized, single-arm, prospective, interventional study, we demonstrate the real-world feasibility and the considerations behind implementing a DL system in prioritizing screening cases according to their likelihood of progression to MOD+ DR.

## Methods

### Inclusion and Exclusion Criteria

The Thailand Prospective Study (TPS) (Thai Clinical Trials Registry #TCTR20190902002) explored the use of Google's automated DR screening software in a real-world clinical setting.^14^ The present nonrandomized, single-arm prospective interventional study, involves 3507 participants, which are a subset of participants in the TPS. Our study was approved by the Office of Research Ethics Committee of Rajavithi Hospital on behalf of all participating sites. Patients gave written informed consent allowing their retinal images to be used in the study.

Five hospitals across Thailand were involved in our study—namely, Phrao, Chomthong, Rajavithi, Khlong Luang, and San Patong Hospitals. Patients underwent a baseline visit as a part of the TPS. The inclusion criteria for TPS were all patients with diabetes in the national diabetes registry above age 18. Exclusion criteria for TPS were patients with a previous diagnosis of DME, severe NPDR, or proliferative DR; prior laser treatment of the retina or retinal surgery; other non-DR eye disease requiring referral to an ophthalmologist; or inability to have fundus photo taken of either eye for any reason. A subset of TPS patients who underwent DR screening between September 2019 and January 2020 and were graded as mild or no DR and not having diabetic macular edema in both eyes were analyzed for our study. A description of the grading approach is described in subsequent paragraphs.

### DL Progression Model

Details of the model have been published previously.^15^ Briefly, the model was based on the Inception-v3 architecture using primary field CFPs, resized to 587 × 587 pixels, as input. The model was developed using data from 289,826 patients from EyePACS Inc., a teleretinal DR screening service in the United States.

The model produces a progression risk score per eye as a number between 0 and 1, representing the likelihood of an eye progressing in terms of DR severity within certain time windows. For this study, the likelihood of progression from no or mild DR to MOD+ within a 1-year window (i.e., at the subsequent annual screen) was used. The model evaluates each eye separately, and the maximum of the two per-eye scores was used for prioritization ranking of patients for this study.

### Generation of Ranked Lists and Clinical Workflow

CFPs from the baseline screening visit were input into the model. A list of deidentified participant IDs, ranked according to progression score output by the model (from highest to lowest) were provided to the Principal Investigator at each site.

Patients were scheduled for their subsequent screening between September 2020 and January 2021 in the proposed order determined by the model from highest to lowest risk. Patients were provided with specific appointment dates by each participating hospital. However, in cases where a patient was unable to accept the given appointment, staff were instructed to find the nearest acceptable appointment day while ensuring that all participants will be screened within 12 to 14 months of their last DR screening, as per the standard of care. Because patients did not always show up on the appointed date, both the scheduled appointment and the actual screening dates were recorded to facilitate analysis. During the visit, clinical staff obtained CFPs and metadata according to routine screening protocol. Fundus images were obtained using Topcon Maestro 3D OCT-1, Topcon TRC NW300, Topcon TRC NW200 and Nidek AFC-210 cameras.

### Grading

Thai retina specialists did the grading of fundus images to obtain the baseline grades of patients with diabetes in the baseline visit (there were two retina specialists, each responsible for a separate set of hospitals assigned to them). After this, fundus images and metadata were transferred to coinvestigators at Google Health via a secure Cloud-based server. A single retina specialist from a pool of six U.S. board-certified retina specialists graded each fundus image for DR and diabetic macular edema to define the subsequent visit grade. This would serve as the progression ground truth for the DL model. Images were ordered randomly and assigned randomly to blind the retina specialist to the model scores. Analysis was performed to compare the DR/diabetic macular edema grades assigned by the retina specialist (progression ground truth) with the ranked list to assess the model's effectiveness in prospectively prioritizing cases.

### Statistical Analysis

Analysis was restricted to those patients with available hemoglobin A1c (HbA1c) results at baseline (because this was a required part of data collection), known follow-up dates, at least one image at the subsequent screen, and known MOD+ outcomes at the subsequent visit. Patients who did not attend within ±60 days of their appointment date, or attended significantly earlier than expected (<150 days since the baseline visit), were excluded from analysis. Images deemed poor quality by the retina specialist were also excluded from analysis. A CONSORT diagram illustrating participant inclusions and exclusions is presented in Supplementary Figure S1.

The analyses were performed by combining patients across sites. The goal of the analysis was to compare the ranking ability of different approaches to scheduling subsequent screenings:

• Approach A: Baseline - ordered randomly
• Approach B: Baseline - all mild DR then all no DR, random order within each group
• Approach C: All mild DR then all no DR, ranked by decreasing HbA1c measurement at baseline within each group
• Approach D: Model proposed order
• Approach E: Observed screening order

Whereas approach A is a simplified approximation to current practice of following a standardized screening interval, approaches B and C represent clinically informed ordering approaches based on available information (DR grade and HbA1c). Approach D represents our DL model, and approach E is the actual order observed in this study. Further detail on how rankings were combined across sites is provided in the Supplementary Methods.

The primary outcome measure was the sensitivity of the model, that is, its ability to rank patients by risk of MOD+ within the first 50% of subsequent screens. This was calculated by dividing the number of patients with MOD+ in the first 50% of rankings by the total number of patients with MOD+ at the subsequent screen.

Approaches A and B were considered the baseline approaches for comparison with approaches C, D, and E. To test the superiority of the model's sensitivity, a permutation test (with 10,000 iterations) was performed under the null hypothesis that the ranking by the model is no different from random ranking (approach D vs A). A second permutation test was performed with the null hypothesis of ranking all mild DR then all no DR, random order within each group (approach D vs B). For each, alpha was set at 0.05. We also report statistical tests comparing approach C and approach E versus both stated null hypotheses in the Supplementary Material.

In addition, we conducted a one-sided Mann–Whitney *U* test for approaches B, C, D, and E to test whether the ranks of the MOD+ progression positives were earlier than the ranks of MOD+ progression negatives. We report the results in Supplementary Table S3.

We devised additional ranking approaches using additional baseline variables, namely, age, duration of diabetes, and insulin use. We first restricted this analysis to a subset of patients where all relevant variables were present, yielding 1646 of 1757 patients. For each baseline variable, we devised a ranking scheme which used the baseline grades to rank the patients, and within those two groups (no DR and mild DR), we ranked the patients using the additional baseline variable. Using the progression ground truth, these rankings were then converted into a plot of fraction screened versus sensitivity plots. For a fair comparison, we also reran the previous approaches (A, B, C, D, and E) on this subset of patients. The results are presented in Supplementary Figure S3.

## Results

A total of 3507 patients who attended their baseline screening with no or mild DR were ranked by the model at five screening sites (Supplementary Fig. S1). Of these, 2365 patients attended a subsequent screening visit. The Chomthong site was excluded from the analysis owing to an incompatible fundus camera used at the subsequent visit and other data inconsistencies. Of the four other sites, four patients who attended unexpectedly soon after the baseline (within 150 days) and four patients who did not attend within 60 days of their appointment date were excluded from the analysis. There were 109 patients who were missing HbA1c data, and they were also excluded from the analysis. In total, 3599 images from 1757 patients were analyzed from four sites.

Demographics and characteristics of analysis-eligible patients are shown in Table. In this cohort of 1757 patients, 63.5% were female. The majority (99.5%) had type 2 diabetes with a mean duration of 7.6 ± 5.9 years. The mean age was 58.7 ± 8.7 years. At baseline, 1578 patients (89.8%) had no DR and 179 (10.2%) had mild DR. The mean time from baseline to subsequent screen was 377.5 ± 37.3 days. At the subsequent screen, 52 (3.0%) patients progressed to MOD+ overall, most of whom (*n* = 37 [71.1%]) attended the Phrao site. At Rajavithi, no patients progressed to MOD+.

**Table.** Demographics and Characteristics of Analyzed Dataset

Study Site	Khlong Luang	Phrao	Rajavithi	San Patong	Total Across 4 Sites
No. of patients	66	1190	55	446	1757
Age, years	60.2 ± 11.1	58.8 ± 8.30	55.9 ± 10.5	58.6 ± 9.01	58.7 ± 8.7
Sex, % female	56.1	68.5	60.0	51.6	63.5
Prediabetes	0	1	0	1	2
Prediabetes duration, years	–	1	–	4	2.5 ± 2.
No. patients, type 1 diabetes	0	5	2	0	7
Type 1 duration, years	–	5.4 ± 4.2	16.0 ± 11.3	–	8.4 ± 7.7
No. patients, type 2 diabetes	66	1184	53	445	1748
Type 2 duration, years	6.8 ± 4.9	7.2 ± 5.8	9.3 ± 7.6	8.7 ± 5.8	7.6 ± 5.9
HbA1c	7.8 ± 1.6	7.3 ± 1.8	7.9 ± 1.6	7.6 ± 1.7	7.4 ± 1.8
Insulin use					
Yes	8	184	16	12	220
No	56	902	39	432	1429
Unknown	2	104	0	2	108
Smoking history					
Yes	3	51	3	13	70
No	60	1137	52	433	1682
Unknown	3	2	0	0	5
Blood pressure, mean (mm Hg)					
Systolic	131.1	128.7	133.0	132.0	129.8
Diastolic	77.1	73	76.9	77.4	74.4
Total cholesterol, mean (mg/dL)	181.0	182.8	170.6	165.3	177.9
No DR at baseline	45 (68.2)	1077 (90.5)	35 (63.6)	421 (94.4)	1578 (89.8)
Mild DR at baseline	21 (31.8)	113 (9.5)	20 (36.4)	25 (5.6)	179 (10.2)
Days between baseline and subsequent visit	353.1 ± 29.4	383.5 ± 36.5	359.1 ± 42.9	367.2 ± 35.3	377.5 ± 37.3
Progressed to MOD+ at subsequent visit	2 (3.0)	37 (3.1)	0 (0.0)	13 (2.9)	52 (3.0)
No DR progressed to MOD+	0 (0.0)	18 (1.7)	0 (0.0)	11 (2.6)	29 (1.8)
Mild DR progressed to MOD+	2 (9.5)	19 (16.8)	0 (0.0)	2 (8.0)	23 (12.8)
Progressed to severe NPDR or above	0	2	0	1	3
Progressed to PDR, number of patients	0	1	0	1	2

HbA1c, hemoglobin A1c.; MOD+, moderate or worse DR; NPDR, nonproliferative DR; PDR, proliferative DR.

Values are mean ± standard deviation, number, or number (%).

### Proposed Versus Observed Order

**Figure 1.**
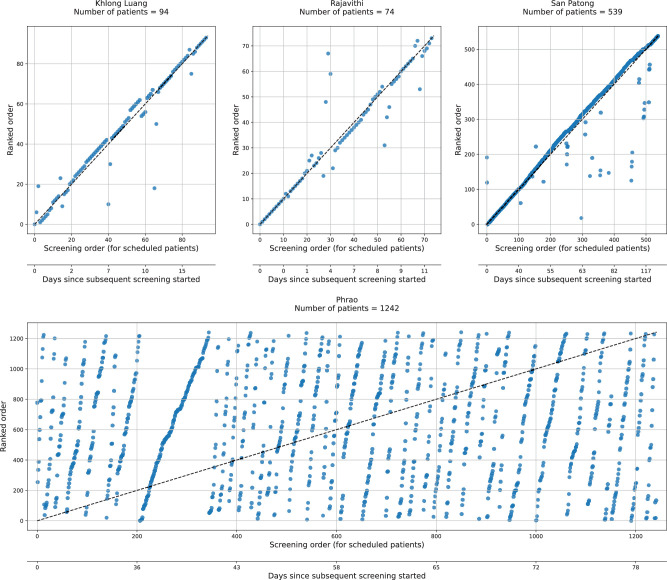
The actual screened order versus the model proposed order (according to the DL progression model), with each patient represented by a dot. Rajavithi, San Patong, and Khlong Luang closely follow the model proposed order, illustrated by a monotonic increasing relationship between the two axes. Phrao followed the order in numerous discrete batches owing to scheduling issues.

The sites attempted to schedule patients in the order proposed by the model (approach D as described in Methods). Three sites (Rajavithi, San Patong, and Khlong Luang) implemented the model proposed order for the majority of patients (Fig. 1). Phrao is a rural center where patients are transported to the hospital in groups via a bus service from their respective locations. However, the bus schedules were nontrivial to modify and resulted in an unanticipated inability to adhere to the ranked order. Excluding this site, the three remaining sites included 567 patients, of which 15 (2.6%) developed MOD+.

### Rate of Progression

The annual transition probabilities between various stages of DR has been studied as part of the assessment of cost effectiveness of screening.^17^^–^^20^ A particularly relevant study by Tung et al.^18^ conducted in Asian patients reported annual transition probabilities that imply a 19.4% and 1.4% rate of progression from no DR and mild DR to MOD+, respectively. By comparison, in our study, the observed numbers were 12.8% and 1.8%, respectively (Table).

### MOD+ Sensitivity Versus Fraction Screened

#### Excluding Phrao

**Figure 2.**
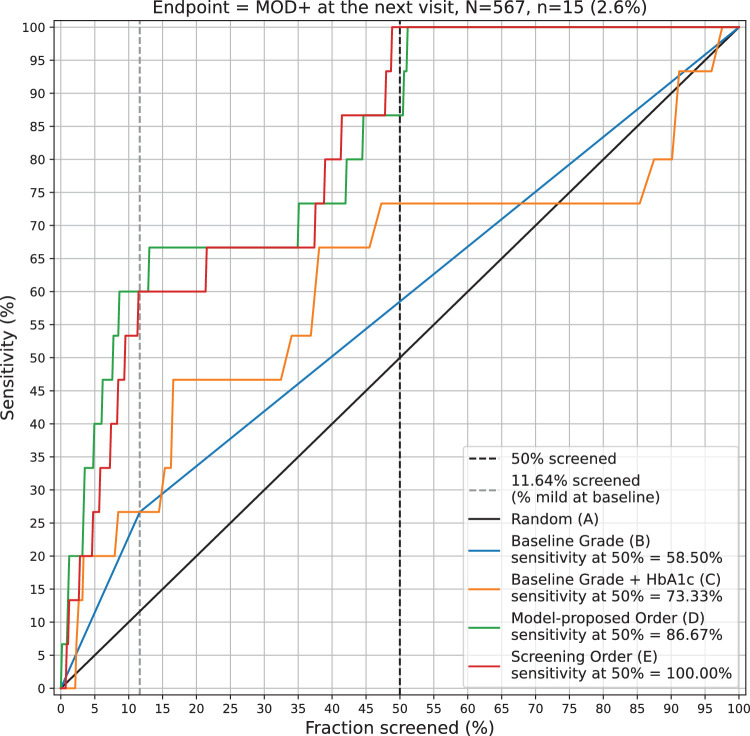
Sensitivity versus fraction screened for Rajavithi, San Patong, and Khlong Luang in aggregate, after excluding Phrao (see Results). The green line represents the model proposed order (approach D), the red line represents the actual observed order (approach E), the blue line represents in order of mild DR and then no DR (approach B), the orange line represents in order of mild/no DR and decreasing HbA1c within each group (approach C), and the black line represents a random order (approach A).

Analysis of the three sites, after excluding Phrao, demonstrates that the sensitivity of the observed screened order (approach E) and model proposed order (approach D) was 100.0% and 86.7%, respectively (Fig. 2). Both of these rankings had superior sensitivity at 50% screened compared with baseline ranking approaches A and B (*P* < 0.001) (Supplementary Table S2). However, because the Phrao site contributed the majority of patients overall as well as the majority of patients that progressed to MOD+ (71% of all patients who developed MOD+), the exclusion of this site decreased dataset size substantially. This study limitation is further addressed in the Discussion.

#### Including Phrao

**Figure 3.**
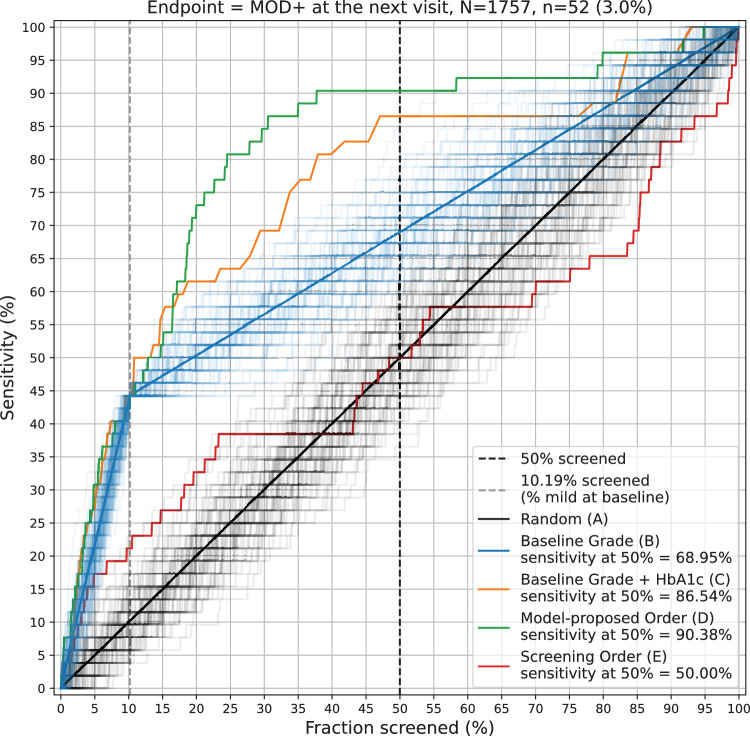
Sensitivity versus fraction screened for Rajavithi, San Patong, Khlong Luang, and Phrao in aggregate (see Results). The green line represents the model proposed order (approach D), the red line represents the actual observed order (approach E), the blue line represents in order of mild DR and then no DR (approach B), the orange line represents in order of mild/no DR and decreasing HbA1c within each group (approach C), and the black line represents a random order (approach A). Each accompanying line for black and blue represents one run of the randomization (which is applicable when there are ties, such as between mild DR patients or between no DR patients). To show the variability that might occur, we plot 100 accompanying lines with a faint color around the main solid line representing the average value.

Using the model proposed order, 90.4% of individuals that developed MOD+ were successfully ranked within the first 50% of subsequent screens (Fig. 3). This was comparable with ranking by mild or no DR and decreasing HbA1c (approach C) which had a sensitivity of 86.5%. The sensitivity at 50% screened using a random order (approach A) and ranking by mild DR then no DR (approach B) was 50.0% and 68.9%, respectively. The model proposed order, and rankings by mild/no DR and HbA1c, had a superior sensitivity to both baseline approaches (approach A and approach B) (*P* < 0.001) (Supplementary Table S1). The percentage of patients graded as mild DR at the baseline screen was 10.2%. At a fraction of 10.2% screened, three ranking approaches (B, C, and D) demonstrated an equal sensitivity of 44.2%. The observed screening order had a sensitivity of 21.1% at this fraction screened. By including Phrao, the observed screening order only achieved a MOD+ detection sensitivity of 50% at 50% fraction screened (Fig. 3), which was not significantly better than random ordering (*P* = 0.49) (Supplementary Table S1).

### Comparison With Alternative Ranking Approaches

We compared three alternative grading approaches on a subset of patients where data was available for relevant baseline variables. Plots can be seen in Supplementary Figure S3. Our proposed approach still outperforms all other approaches, with sensitivity at 50% screened equal to 91.8%. This is closely followed by HbA1c approach (85.7%), followed by duration of diabetes (74.7%), age (71.1%), insulin use (70.5%), and baseline grade alone (68.2%). The random order achieves 50%, and the observed screening order is close behind at 46.9%.

## Discussion

In this prospective study, we demonstrate the capability of our DL model to prioritize diabetic patients for a subsequent screening visit, based on their personalized risk of DR progression. Our evaluation assessed the model's clinical usefulness during COVID-19 recovery efforts in Thailand, with the objective of mitigating delays in scheduling follow-up screening appointments for those at higher risk. The DL model was implemented at sites with high patient volumes, where prioritization of patients based on DR risk could potentially lead to a more efficient allocation of resources and ensure that patients with a higher risk of DR progression receive timely eye clinic visits for appropriate management. Furthermore, our approach directly tackles the problem of optimizing screening intervals by risk stratifying the two largest groups of patients: those without any DR and those with mild DR.

The proposed screening order determined by the DL model (approach D) showed a significantly higher sensitivity (defined as fraction of patients who were MOD+ at second screen who were screened in the first 50% of subsequent screens) compared with baseline methods (approach A and B). The observed screening order (approach E) was highly consistent with the model proposed order at three of the four screening sites, with the exception of the rural Phrao site. At the three consistent-ordering sites (i.e., excluding Phrao), our model successfully provided a more accurate personalized risk assessment to optimize screening intervals. As a result, a greater proportion of patients at higher risk were scheduled for their subsequent annual visit prior to those with lower risk, compared with the baseline methods, ensuring overall earlier detection of progression to MOD+. However, owing to few MOD+ cases at these three sites, there was a lack of power to measure a significant improvement in sensitivity compared with the baseline approaches. Including Phrao in the aggregate analysis, our results showed that simply ordering patients by their baseline grade (mild DR followed by no DR) for the subsequent visit identified 44.3% of those that progressed to MOD+ at 10.2% screened. Although the model proposed order and rankings by grade and HbA1c also demonstrated the same sensitivity at 10.2% screened, both quickly outperformed the baseline approaches as the fraction screened increased, suggesting that both the DL model and HbA1c were good signals for prioritizing patients for subsequent screenings. A notable advantage of the DL model compared with rankings by baseline grade and HbA1c is that it solely requires CFPs (which are already taken for DR screening purposes) as an input, making it a more practical solution for implementation if HbA1c values are not already available. However, in settings where the DL system cannot be deployed, or where HbA1c monitoring is routine, it may be practical and feasible to prioritize subsequent screening visits based on baseline grade and decreasing HbA1c levels.

This study provides data not just on the DL model's performance, but also real-world insights on implementation of the solution. Three of the four evaluated sites successfully implemented the DL model into real-world practice by following the model proposed order for the majority of patients. However, several real-world deployment challenges were encountered during the study. For instance, the attendance order was disrupted by patients who missed their appointment and required rescheduling during the follow-up period. Additionally, patients in certain rural areas traveled to the screening site in groups using prearranged transport, making it challenging to adhere to the model proposed order. This was particularly the case at the Phrao site, which had the greatest number of cases overall and also most of the cases that progressed to MOD+ at the subsequent screen. Because Phrao comprised the majority of patients and MOD+, this factor substantially impacted the sensitivity results using the observed order. Nevertheless, our experience demonstrates the complexities that impact the real-world use of artificial intelligence in health care and highlights the importance of careful evaluation, design, and implementation of systems for improved artificial intelligence delivery.^21^^,^^22^ Implementation science frameworks such as reach, effectiveness, adoption, implementation, and maintenance (RE-AIM) are useful in identifying areas to assess implementation and subsequent dissemination efforts for artificial intelligence in real-world practice.^23^

This work aims to personalize follow-ups according to the patient's risk of progression, which may result in some patients being seen sooner or later than currently recommended guidelines. International guidelines recommend annual DR screening for those without DR or with mild DR^24^^,^^25^; however, the literature suggests that patients without DR could safely undergo screening every 24 months.^26^ Although longer intervals may be sufficient for most patients, a systematic review by Sabanayagam et al.^27^ found that the annual progression from mild to proliferative DR ranged from 0% to 1.5%. Our DL model aims to identify this small proportion of patients who will progress and require more frequent follow-up and may benefit from being prioritized for subsequent screens or being seen sooner than 12 months. This approach has the potential to further decrease the burden of screening on patients and the screening system, and improve efficiency especially in resource-constrained settings.

Limitations of this study include DR grading variability, particularly for subtle retinal changes such as microaneurysms.^28^ Thus, it is possible that a high predicted risk score output by the model may actually indicate existing DR, rather than predicting the future progression of DR. To address this possibility, the concurrent use of a DR grading model with this risk stratification DL progression model could be investigated in future work. Furthermore, images from the baseline and subsequent visits were graded by different retina specialists (the same set of graders were not available for the latter study). Because there can be inter- and intra-rater variability in the assessments, adjudication is generally considered the preferred approach to ensure the accuracy and consistency of the final grading result.^28^ It is also important to note that the DR grades could differ if patients were seen using the proposed order (i.e., the DR grade may differ if screening happened at a different point in time). This means that sensitivity comparisons of the model proposed order with the actual screened order are approximations. This point is particularly relevant for the San Patong site, where subsequent visits were conducted over a period of 4 months. Another limitation is that, given resource constraints on the ground and to limit the burden of data collection by nurses, we were unable to gather data on patient ethnicity, which could have contributed to a better understanding of the patient population and its potential impact on DR progression. Last, because the number of patients who progressed to MOD+ are quite low in our study, we could only compute rough estimates of annual reported DR progression rates.

In conclusion, our study demonstrates the ability of a DL model to help schedule follow-up visits for the two largest subgroups of DR patients based on their risk of progression. To our knowledge, this is the first real-world deployment of a DR progression model in a health care setting. Our findings suggest that such a model could enable earlier identification and treatment of patients at greatest risk of progression before irreversible vision loss occurs. At the same time, it could decrease the burden of screening attendance for lower risk patients by extending their follow-up intervals. However, additional studies are needed to further validate the DL model with a robust dataset for analysis and assess its scalability in screening programs. Further research is needed to evaluate the long-term impact of adapting screening intervals to reflect personalized risk profiles on cost-effectiveness, clinical management, and patient outcomes.

## Supplementary Material

Supplement 1

Supplement 2

Supplement 3

Supplement 1
