# Supplementary material for: Risk Stratification for Diabetic Retinopathy Screening Order Using Deep Learning: A Multicenter Prospective Study
Source: Transl Vis Sci Technol. 2023 Dec 11;12(12):11. doi: 10.1167/tvst.12.12.11 (PMC10715315; doi:10.1167/tvst.12.12.11)
Supplement: Supplement 1 [file tvst-12-12-11_s001.pdf]

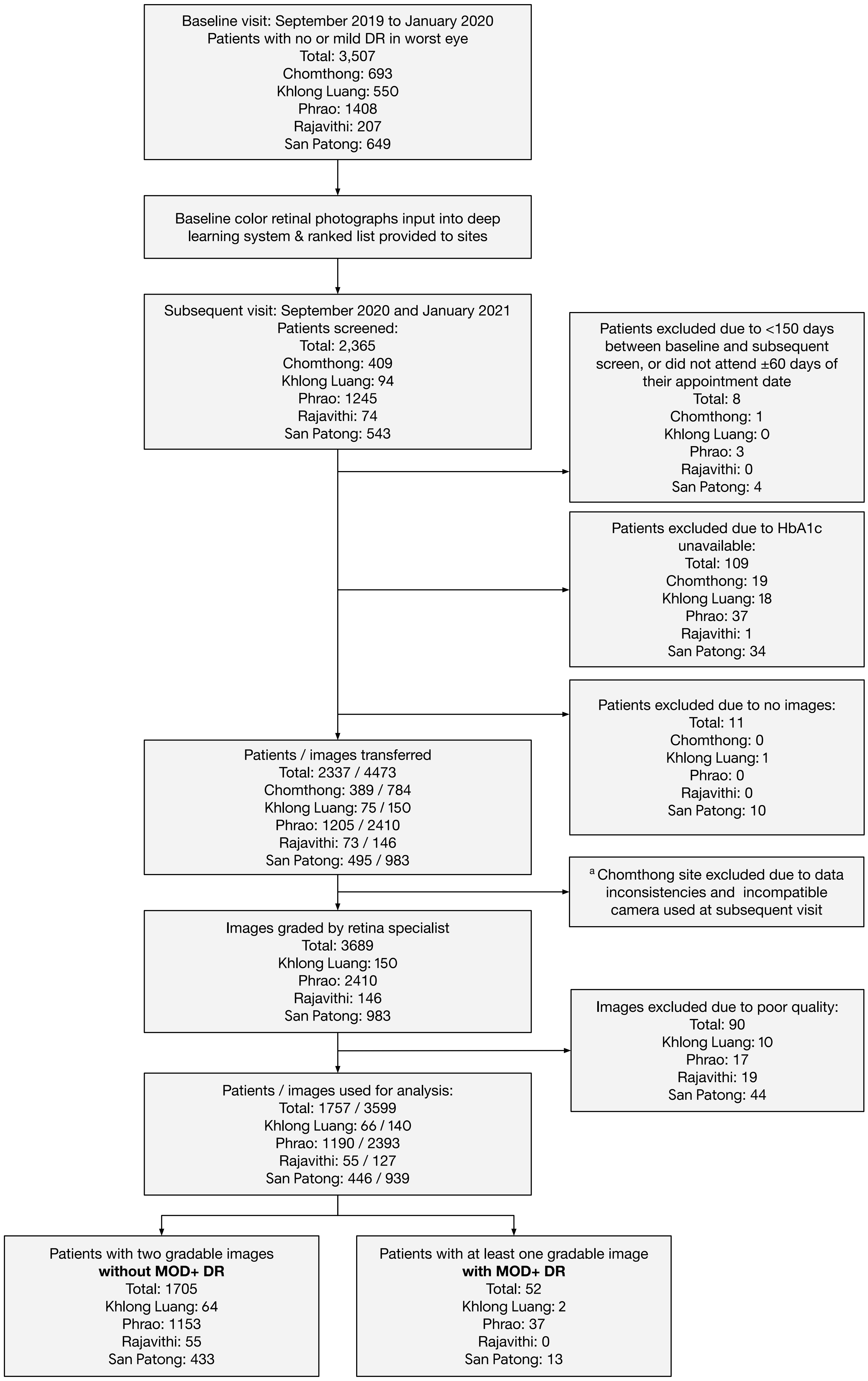

Supplementary Figure 1. CONSORT flow diagram illustrating the participant selection and reasons for exclusions. aThe Chomthong site was excluded from analysis for the following reason: Images for subsequent screenings were captured using a camera with a larger field of view and different contrast from the standard 45-degree camera used at baseline across all sites, and at the subsequent screenings in other sites. As the field of view is known to elicit differences in grading and detection of referable DR, and due to several other inconsistencies identified in the transferred dataset, data from Chomthong was excluded.
