# Supplementary material for: Risk Stratification for Diabetic Retinopathy Screening Order Using Deep Learning: A Multicenter Prospective Study
Source: Transl Vis Sci Technol. 2023 Dec 11;12(12):11. doi: 10.1167/tvst.12.12.11 (PMC10715315; doi:10.1167/tvst.12.12.11)
Supplement: Supplement 2 [file tvst-12-12-11_s002.pdf]

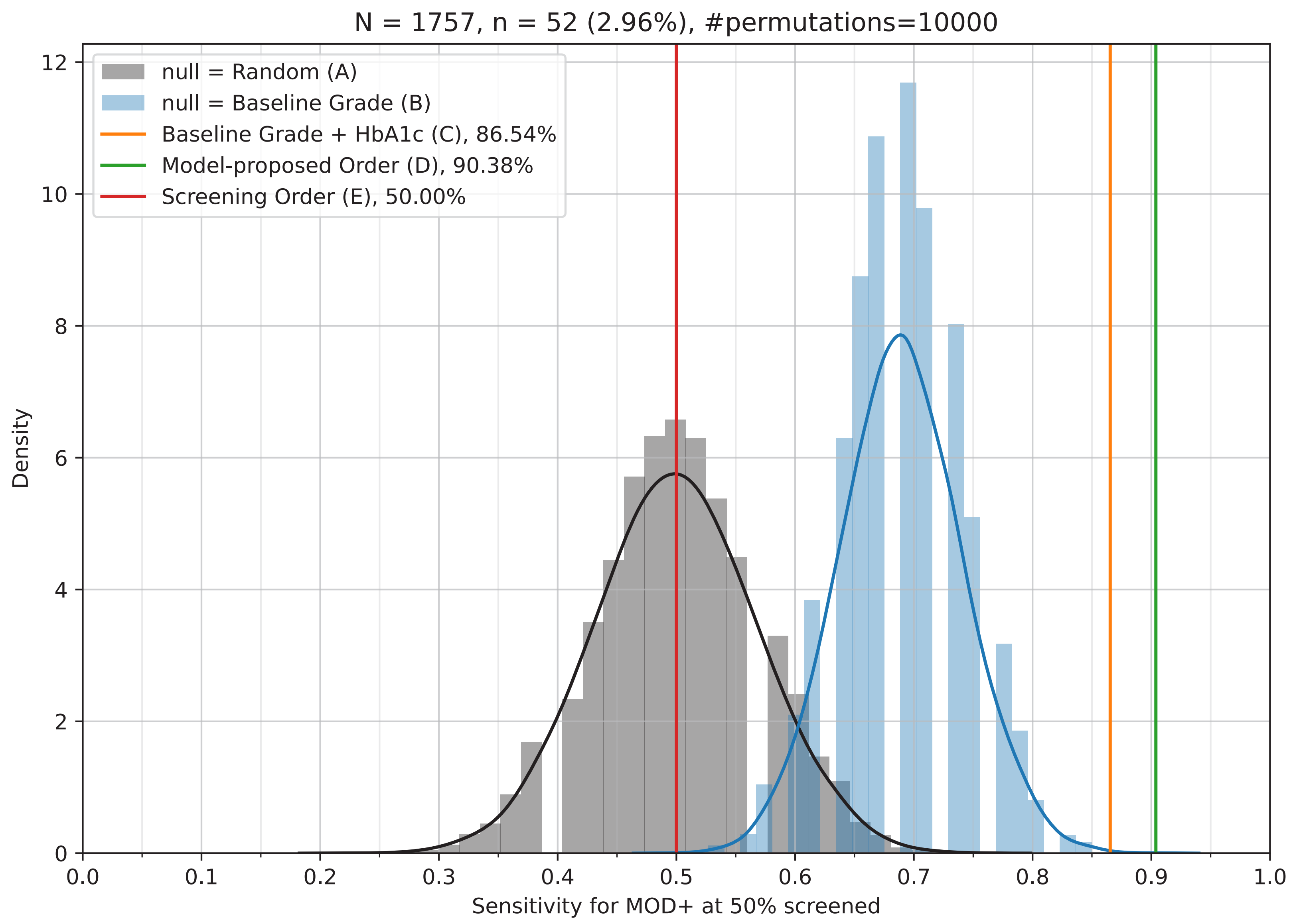

Supplementary Figure 2. Permutation tests for Rajavithi, San Patong, Khlong Luang, and Phrao in aggregate.
