# Supplementary material for: Risk Stratification for Diabetic Retinopathy Screening Order Using Deep Learning: A Multicenter Prospective Study
Source: Transl Vis Sci Technol. 2023 Dec 11;12(12):11. doi: 10.1167/tvst.12.12.11 (PMC10715315; doi:10.1167/tvst.12.12.11)
Supplement: Supplement 3 [file tvst-12-12-11_s003.pdf]

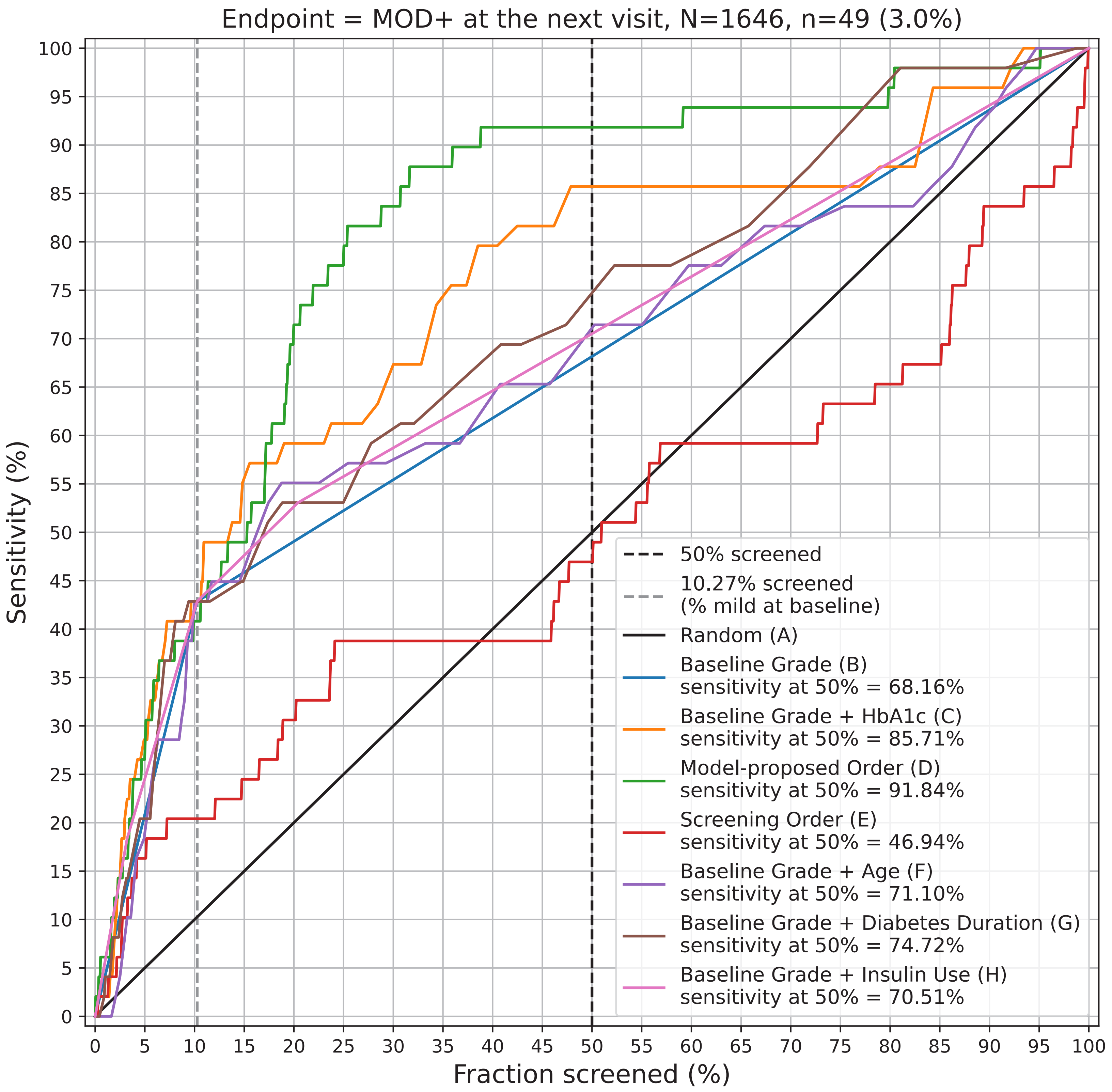

Supplementary Figure 3. Sensitivity versus fraction screened for Rajavithi, San Patong, Khlong Luang, and Phrao in aggregate (see Results) after restricting to patients where age, duration of diabetes, and insulin usage were known. The green line represents the model proposed order (approach D), the red line represents the actual observed order (approach E), the blue line represents in order of mild DR and then no DR (approach B). The orange, purple, brown, and pink lines represent in order of mild/no DR and decreasing values of HbA1c, age, duration of diabetes, and insulin usage within each group, respectively. The black line represents a random order (approach A).
