# Supplementary material for: Risk Stratification for Diabetic Retinopathy Screening Order Using Deep Learning: A Multicenter Prospective Study
Source: Transl Vis Sci Technol. 2023 Dec 11;12(12):11. doi: 10.1167/tvst.12.12.11 (PMC10715315; doi:10.1167/tvst.12.12.11)
Supplement: Supplement 1 [file tvst-12-12-11_s004.docx]

## Supplementary Material

### **Methods**

Since screenings were distributed across multiple sites, we used the following methodology to combine these into a single ranked list to evaluate approach E (observed order). Starting with the model proposed order approach (D), any swap in the order between two patients at the same site were swapped in ranked list D. This approach produces the combined final observed order (E). Another way to think of the same is to take all positions taken by all patients in a particular site in ranking D, doing the permutation from proposed to actual observed order and placing them back. An example is given below:

1. Ranked order (D):

*(siteA, patient1), (siteB, patient2), (siteA, patient3), (siteA, patient4), (siteB, patient5)*

1. Order patients were screened at each site:

*siteA: [patient3, patient4, patient1]*

*siteB: [patient5, patient2]*

1. Combining the sites for the actual observed order (E):

*(siteA, patient3), (siteB, patient5), (siteA, patient4), (siteA, patient1), (siteB, patient2)*

| **Approach** | **Baseline: Random order (A)** | **Baseline: Ordered by mild DR followed by no DR (B)** |
| --- | --- | --- |
| **Baseline Grade + HbA1c (C)** | p < 1e-4 | p = 0.0002 |
| **Model proposed Order (D)** | p < 1e-4 | p < 1e-4 |
| **Screening Order (E)** | p = 0.4941 | p = 1.0000 |

**Supplementary Table 1.** p-values for superiority tests on primary endpoint (sensitivity at 50% screened) at Khlong Luang, Phrao, Rajavithi, San Patong in aggregate.

| **Approach** | **Baseline: Random order (A)** | **Baseline: Ordered by mild DR followed by no DR (B)** |
| --- | --- | --- |
| **Baseline Grade + HbA1c (C)** | p = 0.0372 | p = 0.0923 |
| **Model proposed Order (D)** | p = 0.0016 | p = 0.0057 |
| **Screening Order (E)** | p < 1e-4 | p < 1e-4 |

**Supplementary Table 2.** p-values for superiority tests on primary endpoint (sensitivity at 50% screened) at Khlong Luang, Rajavithi, and San Patong, in aggregate, after excluding Phrao.

| **Approach** | **Sites in aggregate**  **(excluding Phrao)** | **Sites in aggregate**  **(including Phrao)** |
| --- | --- | --- |
| **Baseline Grade (B)** | p = 0.0332 | p < 1e-4 |
| **Baseline Grade + HbA1c (C)** | p = 0.0651 | p < 1e-4 |
| **Model proposed Order (D)** | p < 1e-4 | p < 1e-4 |
| **Screening Order (E)** | p < 1e-4 | p = 0.5495 |

**Supplementary Table 3.** p-values for one-sided Mann-Whitney U test to determine whether the rankings of the MOD+ progression positives were earlier than the rankings of MOD+ progression negatives at all sites in aggregate including Phrao, or excluding Phrao.
